# Evidence Base for the Future of Nursing Homes: Special Issue

**DOI:** 10.1093/geroni/igac039

**Published:** 2022-07-12

**Authors:** Christine E Bishop, Howard B Degenholtz

**Affiliations:** Heller School for Social Policy and Management, Brandeis University, Waltham, Massachusetts, USA; Department of Health Policy and Management, Graduate School of Public Health, Center for Bioethics and Health Law, University of Pittsburgh, Pittsburgh, Pennsylvania, USA

After decades of exposés, special commissions, and advocacy, the moment has arrived to commit to making the residential nursing home a good place to live (National Academies of Sciences, Engineering, and Medicine, 2022). The modern American nursing home, an unexpected creature of the Medicaid and Medicare programs, has evolved to provide neither a comfortable, functional “home” nor reliably excellent “nursing” (Grabowski, 2022). The COVID-19 pandemic has amplified the impact of nursing home shortcomings with respect to safety, clinical quality, racial and ethnic disparities in care, mental health, and resident well-being. The horrendous death toll for nursing home residents and staff, 15.4% of U.S. deaths by April 2022 (Centers for Disease Control and Prevention, 2022; Centers for Medicare and Medicaid Services, 2022), has shocked the nation, as have reports of devastating resident isolation and staff despair.

Sadly, these shortfalls come as no surprise to researchers, advocates, and policy makers who have been grappling with them for decades (Fashaw et al., 2020; Harrington et al., 2019; Hawes et al., 1997; White House, 2022; Wiener et al., 2007). But although previous research has uncovered what does not work in nursing homes, policy and practice have not been able to solve the problems of inconsistent clinical quality, disparities in care, high cost, and poor quality of life. Because these deficits seem so intractable and are sometimes seen as inherent in the nursing home setting itself, avoidance of the nursing home has become the main strategy of policy makers and consumers alike: in partnership with Centers for Medicare and Medicaid Services, state Medicaid programs have worked diligently to “rebalance” toward community living and away from residential long-term services and supports (Bernacet et al., 2021; Eiken et al., 2018; Kaye & Harrington, 2015), and consumers express strong preferences for care in community settings rather than in a nursing home should they become disabled. In a recent survey conducted by AARP, only 2% of people age 50 and older would prefer to receive care in a nursing home (Harrell et al., 2014; see Kasper et al., 2019).

Unfortunately, community living is not optimal for everyone. Given the current state of housing inequity, uncertain capacity of family carers, and home care workforce instability, some individuals with substantial functional limitations are likely to find that a nursing home, or a setting that has many nursing-home-like features, is their best choice (Bishop & Stone, 2014). By shining a spotlight on nursing homes, the pandemic compels us to ask: If we can see what is wrong with nursing homes and accept that we will need nursing-intensive residential services for the foreseeable future, what should be done? The time for simply exposing quality and access problems is long past. Families, payers, and governments must act to make good nursing home care available to those who need it.

This Special Issue of *Innovation in Aging* seizes this pivotal moment. It showcases empirical research for an evidence base to make the nursing home care of the future a preferred setting for older adults with functional deficits. The articles contribute to an agenda for policy and practice to improve the quality of nursing home care. Quality in the nursing home setting is multidimensional, and the articles address quality in several ways. Three articles (Bowblis, 2022; Kishida, 2022; Sharma & Xu, 2022) focus on adequacy and stability of staffing, a basic requirement for structural quality. Another group (Carnahan et al., 2022; Cross & Adler-Milstein, 2022; Davitt & Brown, 2022; Hass et al., 2022; Ninteau & Bishop, 2022) discuss aspects of clinical care that should be targets for improvement and regulation. And a third group (Grabowski, 2022; Morris et al., 2022; Shippee et al., 2022) take us beyond what is currently measured and regulated to investigate factors affecting meaning and quality of life for nursing home residents.

## Managing and Regulating to Support a Stable Nursing Staff

When nursing staff leave or when nursing hours are insufficient, quality suffers. Articles by Kishida, Sharma, and Bowblis and their colleagues provide insights into these issues.

When certified nursing assistants or licensed nurses leave nursing home jobs for positions elsewhere, valuable facility- and resident-specific knowledge is lost. Although nursing homes face the cost of hiring and training replacement workers, the nursing home sector has been living with staff turnover for many years, blaming Medicaid payment constraints for wages that are too low to stem turnover. Both Kishida (2022) and Sharma and Xu (2022) investigate the association of wages, training, and other factors with nursing staff turnover, using data from Japan and Iowa. The Japanese study reports in passing an intriguing policy intervention: bonus payments to nursing homes to be spent directly on training and pay increases. Although low wages are important in retaining direct care workers, both studies find that other unmeasured factors also affect intent to leave. Kishida distinguishes between quitting to take a job in another human services setting and leaving the sector altogether. In the current American labor market, with so many open jobs throughout competing industries, policies to make direct care work more attractive are more important than ever.

The work of the nursing staff becomes next to impossible and quality of care plummets when available staff hours cannot meet resident needs due to turnover, worker shortages, or chronic understaffing. Although a standard for nursing staff adequacy has never been set by regulators, researchers have built up alternative staffing standards from hours of care associated with various resident need characteristics, and the current administration appears ready to codify such a standard (White House, 2022). The article by Bowblis (2022) points out that the majority of U.S. nursing homes staff below and even well below one likely case mix-based staffing standard, and that bringing all nursing homes up to this hypothetical standard would have immense cost, even at current low wage rates.

## Improving Clinical Quality

Beyond the fundamental pressure to assure adequate staffing, some initiatives to sustain nursing home clinical quality look to alignment of ownership incentives with public policy goals, whereas others recommend investments to improve care. Aspects of clinical care that can fall short for residents, including unnecessary hospital transfers, palliative care, and protective services interventions, may require additional infrastructure and new measures for accountability.

The article by Hass et al. (2022) considers the correlations between ownership change and clinical quality measures. Their findings support the growing movement for greater transparency in nursing home ownership. Policies that seek to use value-based payments to drive quality improvement will founder without clarity about how ownership structures can diffuse such incentives.

Residential nursing homes should not attempt to be mini-hospitals, but must still provide a medically safe living environment for residents with complex medical conditions. The article by Carnahan et al. (2022) addresses clinical quality provided at the interface between the nursing home and the hospital. By focusing on the specific issues that make a transfer either necessary or optional, their index potentially increases the probability that a nursing home resident will be treated in place without a disruptive transfer. The next step for this research could be to identify the resources needed to take care of ill nursing home residents in-house. A standardized approach to identifying avoidable hospitalizations has the potential to hold nursing homes accountable for better quality and reduced system cost.

Better information about resident medical care and better coordination across setting could improve clinical care in the nursing home. In an invited essay, Cross and Adler-Milstein (2022) note that nursing homes have been excluded from subsidies that fueled the digital revolution for hospitals and physician practices. Nevertheless, there is evidence that a successful digital transformation would yield substantial benefits in terms of quality of care. It is high time to invest in interoperable resident records, to provide the data needed for medical treatment and personal care whether in the nursing home, in a transfer setting, or after a nursing home stay. Although the value of information sharing may be clearest for Medicare post-acute services, age-friendly coordination of care for long-stay residents would also improve with more shared data.

Ninteau and Bishop (2022) look to pandemic experience to understand the supports nursing staff need in their efforts to relieve concerning symptoms for residents, whether on a day-to-day basis or as part of the dying process. Although attention to resident comfort and care goals is intrinsic to every-day nursing care for this high-need population, end of life care is especially crucial, as even during ordinary times 22% of deaths among people aged 65 and older occur in nursing homes (Centers for Disease Control and Prevention National Center for Health Statistics, 2022). Greater attention to the resources needed for good palliative care, and accountability for its provision, would shore up an important dimension of nursing home quality.

Quality of care is especially challenging to assure for residents who need guardianship protection. When caseworkers could no longer visit clients due to pandemic restrictions, the state of Maryland developed an initiative to provide communication through technology. Davitt and Brown (2022) describe its implementation and use and note that individualized digital equipment could also foster communication and social engagement for residents not in need of state guardianship. This case study provides another example of innovation spurred by pandemic necessities that may have broader implications.

## Advancing Equitable Access to Resident Quality of Life

The nursing home of the future must be resident centered, equitable, and focused on quality of life even as it meets clinical quality standards. Despite the expansion of the quality survey process to include resident voices, aspects of nursing home care that could support well-being and community engagement have not been sufficiently addressed by nursing homes, their funders, or their regulators. Articles in this Special Issue target the shortcomings of dementia care, inequities in quality of life, and the importance of resident autonomy.

Morris et al. (2022) jolt our thinking by labeling some aspects of current dementia care practices as iatrogenic. Their analysis exposes the violence to personhood inherent in the physical and psychological staff tactics meant to protect and contain reactive residents with cognitive impairments. The conceptual frame implies raising the standard for day-to-day care for the growing proportion of nursing home residents experiencing cognitive deficits. Moving forward, insights from this thoughtful article should inform nursing staff training and regulation alike.

A deeply informative mixed-methods pilot study by Shippee et al. (2022) focuses on quality of life for nursing home residents of color. The authors were able to contrast nursing homes with little disparity in quality of life with those where non-White residents experienced substantially lower quality of life. In-depth interview data from multiple stakeholders uncovered possible drivers of these differences. The good news is that in facilities with the greatest disparity between White and non-White residents, researchers could identify potentially modifiable processes apparently responsible for quality of life inequities. Training and diversifying leadership and staff, especially activities staff, could help, as might fostering engagement from a range of community organizations preferred by the residents. There are broader implications here: too little is known about how good quality of life can be monitored and supported for all nursing home residents, and how nursing homes can be held accountable for providing it.

Finally, David Grabowski’s invited essay (2022) considers the larger issues facing nursing home policy and follows on from Shippee et al. and others in this Special Issue by articulating an overarching goal for transforming nursing home care: residents are owed environments that are first and foremost resident centered. Grabowski’s summary article argues convincingly that performance can be linked to payment policy, concluding that policy makers should focus on reforms that improve both payment and accountability, with resident centeredness as the ultimate goal.

## Conclusion and Next Steps

The body of research collected in this Special Issue suggests some solutions and future directions for policy makers, providers, and researchers. The articles open many new questions and creative concepts. We invite our readers to explore and be inspired to rededicate themselves to improving the quality of life and quality of care for people living in nursing homes.

## Conflict of Interest

None declared.
